# Associations Between IT Job Stressors and Anxiety, Depression, and Stress: Cross-Sectional Study

**DOI:** 10.2196/73211

**Published:** 2026-03-03

**Authors:** Edlin Garcia Colato, Nianjun Liu, Angela Chow, Catherine M Sherwood-Laughlin, Jonathan T Macy

**Affiliations:** 1 Department of Health and Wellness Design, School of Public Health, Indiana University Bloomington, 1025 E 7th St, Bloomington, IN, 47405, United States, 1 812-855-3102; 2 Department of Epidemiology and Biostatistics, School of Public Health, Indiana University Bloomington, Bloomington, IN, United States; 3 Department of Applied Health Science, School of Public Health, Indiana University Bloomington, Bloomington, IN, United States

**Keywords:** survey, association, occupational health, mental health, stressors, IT, IT professionals, United States, workplace, depression, anxiety, stress, help-seeking, health literacy

## Abstract

**Background:**

The IT sector is growing and encompasses all professions, from leisure and recreation to hospitals and emergency response groups. IT professionals are experiencing increased threats (eg, ransomware attacks), but little is known about the relationship between these IT profession–specific stressors and the professionals’ mental health.

**Objective:**

This study aimed to (1) estimate the associations between IT profession–specific stressors and anxiety, depression, and stress, and (2) examine the role of mental health literacy (MHL) as a mediator of the relationship between depression, anxiety, stress, and help-seeking.

**Methods:**

Between February and May 2023, IT professionals working in the United States were surveyed online. Participants (n=357) reported demographic characteristics, MHL, mental health symptoms, and help-seeking intentions with the following scales: Mental Health Literacy in the Workplace (MHL-W), Center for Epidemiological Studies Depression-10 (CESD-10), Generalized Anxiety Disorder-7 (GAD-7), Perceived Stress Scale-10 (PSS-10), and the Mental Help Seeking Intention Scale (MHSIS). Descriptive statistics, regression models, and mediation analyses were conducted for CESD-10, GAD-7, and PSS-10.

**Results:**

Respondents who had experienced ransomware attacks in the past year reported significantly higher symptoms of depression (odds ratio [OR] 1.85, 95% CI 1.07-3.22; *P*=.03). Past-year exposure to balancing security and usability was associated with lower odds of reported anxiety (OR 0.48, 95% CI 0.28-0.82; *P*=.008). Having made critical technology decisions with limited information in the past year was associated with higher perceived stress by 2.02 points on the PSS-10 scale (SE 0.84, 95% CI 0.37-3.66; *P*=.02), and working with limited resources in the past year increased perceived stress by 1.70 points (SE 0.84, 95% CI 0.04-3.35; *P*=.04) after adjusting for the covariates. MHL was found to partially mediate the relationship between depression and help-seeking, but not between anxiety or stress and help-seeking.

**Conclusions:**

These findings provide insight into the workplace stressors that pose a greater psychological health risk for IT professionals. These results emphasize the important role of MHL in helping facilitate the connection between depressive symptoms and help-seeking.

## Introduction

Established in the 1950s, IT is defined as the “use of computer systems or devices to access information” for both business and personal operations, such as “storing, retrieving, accessing or manipulating information” [1]. The efforts of IT workers to maintain business devices can sometimes involve high-stress exposures such as viewing illicit content and mitigating ransomware attacks [2]. Technologies have become persistent targets for hackers and cybercriminals who seek vulnerabilities in networks [3]. Ensuring the safekeeping of technology means that some IT professionals are in a constant state of high alert. The high levels of stress experienced by IT workers are reflected in the findings from a recent assessment of challenges and stressors in the information security sector. According to the 2022‐2023 Chartered Institute of Information Security report, 22% of 302 UK respondents reported working more than 48 hours per week, with 8% working more than 55 hours weekly [4]. Furthermore, 32% reported they were kept awake by worries of a potential cyber-attack on their organization, up from 22% in 2022 [5]. Over two-thirds of the survey respondents believed there would be an increase in the frequency and impact of ransomware attacks [5].

The existing literature on mental health in IT has largely focused on IT professionals working in Asia [6,7] and Europe [5,8]. Following a ransomware attack, Northwave, a security company based in the Netherlands, conducted a study with 21 members of its own computer emergency response team employees and found that mental health can be significantly affected by ransomware attacks [9]. While the Northwave study suggested an elevated risk of mental health issues among IT professionals, another study using the UK Biobank cohort study did not yield similar findings [10]. This was the first UK-based longitudinal study (running from 2006 to 2010); it compared the incidence of anxiety and depression between IT and non-IT employees aged 40 years or older found that IT professionals had a reduced risk of anxiety and depression compared to their non-IT counterparts [10]. To the best of our knowledge, no such report exists for the IT sector in the United States.

Considering the IT professionals who report experiencing symptoms of depression, anxiety, and stress, an important next step is to determine whether these individuals possess a firm knowledge base on mental health and whether they intend to seek help. Mental health literacy (MHL) refers to the knowledge and ability to recognize and identify symptoms related to mental health for preventing mental illness as well as maintaining and promoting mental health [11]. Previous research has found a positive correlation between MHL and mental health attitudes [12,13], and young adults with more favorable attitudes toward mental health services are more likely to seek help [14].

Past studies have not assessed MHL, mental health attitudes, or intention to seek help among IT workers in the United States. Additionally, previous studies have not considered important factors such as exposure to illicit content, ransomware attacks, hacking, and takedowns, which may contribute to the elevated symptoms of depression, anxiety, and stress experienced by IT professionals. A recently published occasional paper from the United Kingdom examined first-order harms of ransomware on staff and identified the stress reported by incident responders following the incident [15]. First-order harms are “harms to any organization and their staff directly targeted by a ransomware incident” [4]. There is an urgent need for evidence-based resources and information concerning mental health in the IT workforce community, a domain that remains under-studied, especially in the US context. Therefore, this study aimed to (1) test the relationship between IT profession–specific stressors and anxiety, depression, and stress and (2) analyze the role of MHL as a mediator for the 3 selected mental health conditions (anxiety, depression, and stress) and help-seeking behaviors.

## Methods

### Participants and Data Collection

Data for this study were collected between February and May 2023 via an online survey using Qualtrics [16]. The online open survey was developed with previously validated scales described in detail in the Measures section below. For this convenience sample, 2336 participants were identified via known contacts, SurveyCircle [17], and Prolific [18]. The three eligibility criteria for the survey were (1) being aged at least 18 years, (2) working in the IT sector in the United States, and (3) having at least 12 months of any IT work experience. Electronic written voluntary consent was recorded for all survey respondents at the start of the survey. Duplicate entries were avoided by limiting access to the survey to a single attempt. Of the original 483 responses recorded, 23 observations were excluded because of either discontinuing the survey prior to providing consent, being ineligible, or being a bot entry. Review of the data showed no evidence of conspicuous response behavior. Nevertheless, outliers in average completion time of the survey, such as those showing that the survey was completed in only a few minutes, were excluded. A total of 388 (84.3%) of the remaining 460 participants who provided consent and whose responses were determined to be valid completed the survey. The final sample following complete case handling of missing data is described below in the Data Analysis section.

### Ethical Considerations

The consent form included pertinent information, such as the estimated duration of the survey, the fact that personally identifiable information would not be collected, and the purpose of the study. After respondents completed the 10-to-15-minute survey, they were given the option to take part in a drawing for one of four US $75 Target e-gift cards. To ensure anonymity in the survey responses, respondents were guided to a separate Qualtrics survey where they could provide an email address for the random drawing. This study received approval as an exempt study by the Indiana University Bloomington Human Subjects and Institutional Review Board (protocol 18281).

### Measures

#### Assessment of Mental Health Status

Symptoms of depression were assessed with the 10-item Center for Epidemiologic Studies Depression (CESD-10) questionnaire [19,20]. The CESD-10 helps identify individuals at risk of developing clinical depression and has been previously validated against the original 20-item CESD questionnaire [21] designed for screening the general population [19,20,22]. CESD-10 scores range between 0 and 30; scores below 10 are recoded to 0 (“no significant symptoms of depression”) and scores 10 and above are recoded to 1 (“significant symptoms of depression”) creating a binary variable for depression.

Meanwhile, the 7-item Generalized Anxiety Disorder Scale (GAD-7) was used to screen for symptoms of anxiety among the respondents [23]. Responses to the 7 questions were summed for a final score ranging from 0 to 21. For descriptive purposes, 3 cutoff points were used (5, 10, and 15; 0‐4=minimal anxiety, 5‐9=mild anxiety, 10‐14=moderate anxiety, and 15‐21=severe anxiety) to show the different categorical levels of severity. However, the optimal cutoff score for screening anxiety via the GAD-7 scale is 10 [23,24]. Therefore, scores 10 and above were coded as 1 (“yes”) for anxiety and below 10 were coded as 0 (“no/minimal”) for level of anxiety.

Unlike the anxiety and depression scales, the 10-item Perceived Stress Scale (PSS-10) does not translate to clinical significance; therefore, categorizing scores into groups is done only for descriptive purposes, and for the analyses the scores were included as a continuous variable [25]. Final scores ranged between 0 and 40. For descriptive purposes, scores 0 to 13 were coded as “low stress,” 14 to 26 as “moderate stress,” and scores 27 to 40 as “high stress.” Increased perceived stress is reflected by higher scores.

#### IT Profession–Specific Stressors

For research question 1, we identified 12 past-year IT profession–specific stressors based on the feedback solicited from 2 IT professionals with a combined 35 years of IT experience. IT experts were selected based on predefined criteria, including their professional qualifications and practical experience. Interviews were conducted with the IT experts, allowing for the development of a comprehensive list of stressors. The list of the 12 stressors curated by the IT professionals was as follows: (1) ransomware attacks, (2) exposure to illicit content, (3) takedowns, (4) handling sensitive data and cybersecurity threats, (5) making critical technology decisions with limited information, 6) adapting to rapid changes in technology and business requirements, (7) pressure to solve complex technical issues, (8) the constant need to stay up to date with technology, (9) dealing with unexpected system failures and outages, (10) dealing with leadership that does not wish to invest in or be inconvenienced by cybersecurity initiatives, (11) balancing security and usability, and (12) working with limited resources (eg, budget and personnel).

Respondents were asked a single question: “Which of the following on-the-job stressors have you experienced in the past year?” and selected all that applied from the list of stressors provided. The 12 stressors were measured individually as binary variables (yes/no), and a separate count variable was created to identify the total number of the 12 stressors experienced by each respondent (scores ranged from 0 to 12).

#### Mental Health Help-Seeking Intentions

For research question 2, the primary outcome was mental health help-seeking intentions, which were measured using the Mental Help Seeking Intention Scale (MHSIS) [26]. Final MHSIS scores ranged between 1 and 7. Higher scores indicate greater intention to seek help from a mental health professional [26].

#### Mental Health Literacy in the Workplace

The Mental Health Literacy Tool for the Workplace (MHL-W) comprises 4 vignettes depicting a hypothetical coworker’s behavior in the workplace; each vignette has 4 questions measuring 4 distinct MHL concepts [27]. The following 4 variables were rated on a scale from 1 (very low) to 5 (very high): (1) level of knowledge in being able to recognize a specific disorder (“What might be happening with [him/her]),” (2) level of knowledge and beliefs regarding risk factors and prevention (“How you could prevent the situation from becoming worse”), (3) level of knowledge and attitudes about help-seeking (“What you should say or do in the situation”), and (4) level of knowledge and beliefs regarding interventions (“Resources or services that might be helpful”). The values for all 16 questions were summed up for a final MHL-W score, with possible scores ranging from 16 to 80. Higher scores indicate a greater self-reported knowledge of mental health.

#### Demographics

Demographic data were collected on the following characteristics: age, race (Black or African American, American Indian or Alaska Native, Asian or Asian American, Native Hawaiian or other Pacific Islander, White, and other), ethnicity, sex at birth (female vs male), relationship status, education, and income-level groups. Data regarding health-related characteristics included questions about health insurance and mental health history.

#### Covariates

Covariates included age, sex, race, ethnicity, education, income, health insurance, and mental health history.

### Data Analysis

Descriptive statistics, means and SD for continuous variables, and frequencies and percentages for categorical variables were computed by respondent sex at birth. A 2-sided *P* value of less than .05 was considered significant. A complete case analysis was performed, excluding participants with any missing data for key variables. Of the 388 participants, 17 (4%) were missing data for age, 1 (<1%) for race, 4 (1%) for ethnicity, 1 (<1%) for education, 2 (<1%) for health insurance, 5 (1%) for CESD-10, 1 (<1%) for GAD-7, 13 (3%) for PSS-10, 1 (<1%) for MHSIS, and 10 (3%) for MHL-W. A total of 357 participants had complete data on all key variables.

The following models were used to address the first hypothesis and identify which IT profession–specific stressors were associated with anxiety, depression, and stress: (1) unadjusted and adjusted logistic regression models to examine the relationship between each of the 12 IT-profession specific stressors and depression (CESD-10), (2) unadjusted and predictor-adjusted logistic regression models to test for the relationship between each of the 12 IT-profession specific stressors and anxiety (GAD-7), and (3) unadjusted and predictor-adjusted linear regression models to test for the association between each of the 12 IT-specific stressors and stress (PSS-10).

A confirmatory factor analysis (CFA) was conducted for the MHL-W because the workplace questionnaire had previously been used only in a single workplace setting in health care. The CFA aimed to test whether each of the 4 questions per vignette loaded onto the intended corresponding MHL constructs.

### Mediation Analysis

Mediation analysis was conducted using the *medsem* package in Stata (StataSE version 17.0; StataCorp). Outputs for the structural equation modeling for the mediation analysis included a modified version of the Sobel test for assessing indirect effects [28], as well as the Monte Carlo resampling approach for assessing indirect effects [29]. Descriptive statistics, a CFA, mediation, and all other analyses were conducted using Stata.

## Results

### Participants

**Figure 1.**
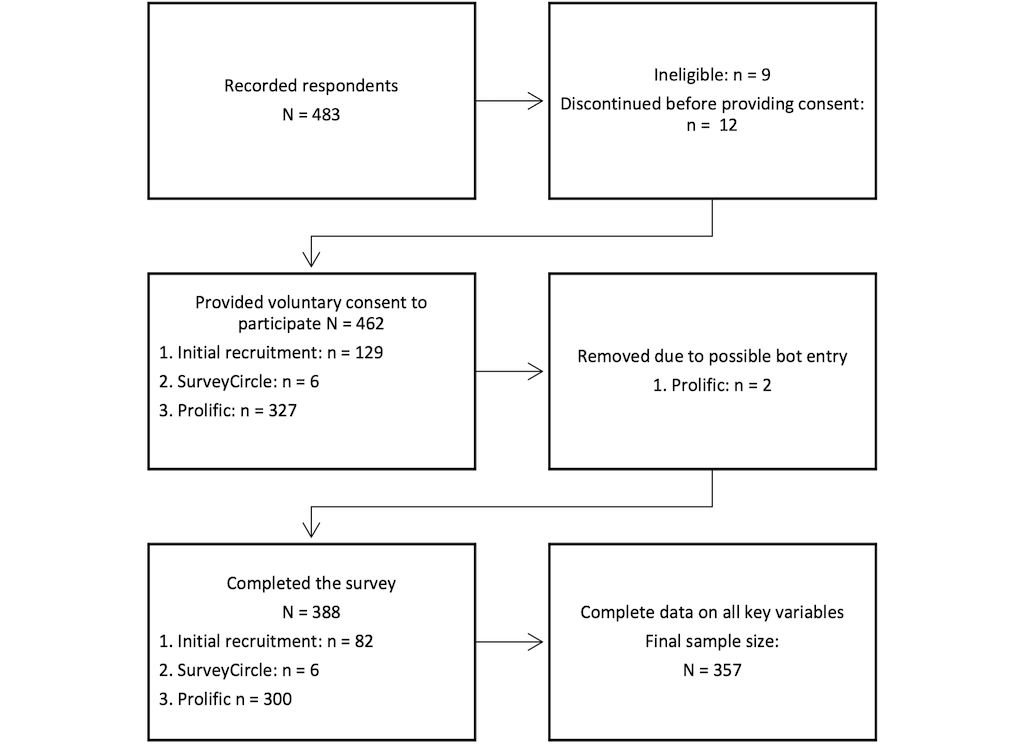
Flow diagram of the study sample.

Figure 1 shows a flowchart of participation, and Table 1 shows participant characteristics. The majority of the 357 respondents self-identified as male (n=264; 73.9%), were White (n=281; 78.7%), were of non-Hispanic/Latinx ethnicity (n=323; 90.5%), had earned a bachelor’s degree (n=181; 50.7%), and earned US $75,000 or more a year (n=213; 59.7%).

**Table 1.** Participant characteristics by sex (n=357).

		Female (n=93; 26.1%)	Male (n=264; 73.9%)	Total
Age (years), median (SD)		42 (11.4)	39 (10.7)	39 (10.9)
Race, n (%)				
	African American or Black	4 (4.3)	12 (4.5)	16 (4.5)
	American Indian or Alaskan Native	0 (0.0)	3 (1.1)	3 (0.8)
	Asian or Asian American	12 (12.9)	31 (11.7)	43 (12.0)
	White	74 (79.6)	207 (78.4)	281 (78.7)
	Other	1 (1.1)	3 (1.1)	4 (1.1)
	Two or more selected	2 (2.2)	8 (3.0)	10 (2.8)
Ethnicity, n (%)				
	Non-Hispanic	82 (88.2)	241 (91.3)	323 (90.5)
	Hispanic	11 (11.8)	23 (8.7)	34 (9.5)
Education, n (%)				
	Less than a bachelor’s degree	22 (237)	83 (31.4)	105 (29.4)
	Bachelor’s degree	43 (46.2)	138 (52.3)	181 (50.7)
	Graduate’s degree	28 (30.1)	43 (16.3)	71 (19.9)
Health insurance type, n (%)				
	Employer provided	61 (65.6)	176 (66.7)	237 (66.4)
	Government provided	27 (29.0)	78 (29.5)	105 (29.4)
	None	5 (5.4)	10 (3.8)	15 (4.2)
Income group (US $), n (%)				
	Less than 50,000	18 (19.4)	52 (19.7)	70 (19.6)
	50,000-74,999	20 (21.5)	54 (20.5)	74 (20.7)
	75,000-99,999	23 (24.7)	48 (18.2)	71 (19.9)
	≥100,000	32 (34.4)	110 (41.7)	142 (39.8)
Mental health history, n (%)				
	Yes	42 (45.2)	57 (21.6)	99 (27.7)
	No	51 (54.8)	207 (78.4)	258 (72.3)
CESD-10^a^ score, n (%)				
	No/low	50 (53.8)	181 (68.6)	231 (64.7)
	Moderate/severe	43 (46.2)	83 (31.4)	126 (35.3)
GAD-7^b^ score, n (%)				
	No/low	72 (77.4)	215 (81.4)	287 (80.4)
	Moderate/severe	21 (22.6)	49 (18.6)	70 (19.6)
MHSIS^c^ score, mean (SD)		5.33 (1.5)	5.11 (1.7)	5.17 (1.7)
MHL-W^d^ score, mean (SD)		47.95 (11.9)	46.46 (10.9)	46.85 (11.2)
PSS-10^e^ score, mean (SD)		17.15 (7.9)	14.30 (8.1)	15.04 (8.1)

a CESD-10: 10-item Center for Epidemiologic Studies Depression Scale.

b GAD-7: 7-item Generalized Anxiety Disorder.

c MHSIS: Mental Help Seeking Intention Scale.

d MHL-W: Mental Health Literacy at the Workplace.

e PSS-10: 10-item Perceived Stress Scale.

### Outcome Data

The majority of participants (231/357, 64.7%) were below the minimum cutoff score of 10 for depression as assessed by the CESD-10. Similarly, 287 of 357 (80.4%) respondents reported having no or low symptoms of anxiety. The mean PSS-10 score was 15.04 (SD 8.1), representing a moderate level of stress. For mental health history, most (258/357, 72.3%) of the respondents reported having no known previous mental health diagnosis. On average, 5 stressors were experienced in this population, as shown in Table S1 in Multimedia Appendix 1. Past-year takedowns were experienced the least (75/357, 21.1%) by the sample, while experiencing pressure to solve complex technical issues was the most common experience (252/357, 70.1%).

### Main Results

#### IT Profession–Specific Stressors and Depression

Prior to controlling for sociodemographic variables, none of the stressors were significantly associated with depression. The respondents who reported past-year exposure to ransomware (odds ratio [OR] 1.85, 95% CI 1.07-3.22; *P*=.03) or working with leadership that did not wish to invest in or be inconvenienced by cybersecurity initiatives (OR 1.75, 95% CI 1.01-2.97; *P*=.048) in the past year were more likely to have significant symptoms of depression, after controlling for the covariates. The total number of stressors experienced in the past year also had no significant association with depressive symptoms, even after controlling for the covariates. None of the remaining IT profession–specific stressors experienced in the past year were individually associated with significant symptoms of depression.

#### IT Profession–Specific Stressors and Anxiety

Past-year exposure to balancing security and usability was associated with lower odds of reported anxiety (OR 0.48, 95% CI 0.28-0.82; *P*=.008); however, that association did not hold once adjusting for the covariates. All remaining IT stressors had no significant association with anxiety, including after controlling for the covariates.

#### IT Profession–Specific Stressors and Stress

Controlling for the covariates, respondents who reported making critical technology decisions with limited information in the past year had higher perceived stress by 2.02 points on the PSS-10 scale (SE 0.84, 95% CI 0.37-3.66; *P*=.02). Similarly, respondents who reported working with limited resources (eg, budget and personnel) in the past year had higher perceived stress by 1.70 points on the PSS-10 scale (SE 0.84, 95% CI 0.04-3.35; *P*=.04), after adjusting for the covariates. The remaining stressors did not have a significant relationship with perceived stress.

#### Mediation Analysis

Mediation analysis was performed to assess the mediating role of MHL-W score in the relationship between depression, anxiety, and stress scores (separately) and help-seeking intentions (MHSIS). The results (shown in Table 2) revealed that depression also significantly predicted MHL-W score (coefficient=–2.236; *P*=.049) and that MHL-W score significantly predicted MHSIS score (coefficient=0.037; *P*<.001). After controlling for MHL-W score, the direct effect of depression on MHSIS score remained significant (coefficient=–0.438; *P*=.01). Per the Sobel test and the Monte Carlo approaches, MHL-W score partially mediated the relationship between depression and help-seeking intentions.

**Table 2.** Mediation results for mental health literacy and depression, anxiety, and stress.

Variable and effect type		Coefficient	Z value	*P* value	95% CI
CESD-10^a^ → MHL-W^e^ → MHSIS^f^					
	Total effect (CESD-10 → MHSIS)	–0.524	—^d^	—	^—^
	Direct effects (CESD-10 → MHSIS)	–0.438	–2.53	.01	–0.778 to –0.098
	Indirect effects of CESD-10 on MHSIS	–0.086	–1.826	.07	–0.194 to –0.003
GAD-7^b^ → MHL-W → MHSIS					
	Total effect (GAD-7 → MHSIS)	0.033	—	—	—
	Direct effects (GAD-7 → MHSIS)	–0.517	–2.49	.01	–0.924 to –0.110
	Indirect effects of GAD-7 on MHSIS	–0.018	–0.31	.75	–0.128 to 0.093
PSS-10^c^ → MHL-W → MHSIS					
	Total effect (PSS-10→ MHSIS)	–0.048	—	—	—
	Direct effects (PSS-10→ MHSIS)	–0.028	–2.83	.005	–0.048 to –0.009
	Indirect effects of PSS-10 on MHSIS	–0.005	0.003	.13	–0.011 to 0.001

a CESD-10: 10-item Center for Epidemiological Studies Depression.

b GAD-7: 7-item Generalized Anxiety Disorder.

c PSS-10: 10-item Perceived Stress Scale.

d Not applicable.

e MHL-W: Mental Health Literacy in the Workplace.

f MHSIS: Mental Help Seeking Intention Scale.

On the contrary, the results for mediation analyses for anxiety and stress did not support MHL-W score as a mediator for anxiety or stress and help-seeking intentions. Although there was a significant relationship between MHL-W score and MHSIS score, there was no significant relationship between GAD-7 score or PSS-10 score and MHL-W score.

## Discussion

### Key Results

This study was conducted to (1) test which IT profession–specific stressors were associated with depression, anxiety, and stress; and (2) examine the impact of depression, anxiety, and stress on help-seeking as mediated by mental health literacy. For the first objective, we found that past exposure to ransomware attacks and working with leadership that did not wish to invest in or were inconvenienced by cybersecurity initiatives were both associated with higher odds of depressive symptoms. While none of the stressors had a significant relationship with anxiety, two stressors did have a significant relationship with perceived stress. Making critical technology decisions with limited information and working with limited resources were both linked to higher perceived stress. Regarding the second objective, we found that mental health literacy in the workplace only partially mediated the relationship between depression and help-seeking intentions, but not between anxiety or stress and help-seeking intentions.

The findings for the first objective align with and complement findings from prior work. For instance, Northwave’s report on the psychological impact of a ransomware attack on their employees showed there was significant stress experienced immediately after the incident and throughout the following year [9], while in this study, ransomware was associated with symptoms of depression and not current perceived stress. This might be because the stress instrument used captures general stress over the past month and is not event-specific. However, the depression questionnaire used captured longer-term emotional stress, which develops over time after prolonged exposure to high-stakes incidents (eg, ransomware). Furthermore, having a leadership team that is unsupportive of cybersecurity initiatives, which would help deter ransomware attacks, was also associated with increased depressive symptoms. This adds to the ransomware literature, which is heavily focused on the financial impact on individuals and organizations and the psychosocial costs to the victims, rather than considering the individuals working tirelessly to mitigate the problem [30,31]. Only two stressors had a significant association with increased perceived stress: making critical decisions with limited information and working with limited resources. Both of these stressors and increased stress align with the job demands–resources model, in which having inadequate resources places strain on the worker, increasing risk of burnout [32,33].

The pressure to solve complex technical issues, the constant need to stay up to date with technology, and the task of dealing with unexpected system failures and outages were not associated with depression, anxiety, or stress. This is likely because these stressors, except for the unexpected system failures and outages, are typical daily job activities and expected tasks and responsibilities for individuals working in the IT sector. Staying up to date with technology and solving complex technical issues are part of the allure of working in technology. These may act as positive stressors, potentially increasing stress while also promoting professional and personal growth, rather than predominantly negative experiences for IT professionals [34]. The influence of positive and welcomed anticipated stressors among IT professionals requires further exploration.

Interestingly, exposure to illicit content was not associated with depression, anxiety, or stress in this study. Previous literature has found that exposure to illicit content (eg, exploitative, violent, and/or abusive content) can have psychologically detrimental effects on children and adolescents [35]. While similar associations have not been extensively documented among adults, Federal Bureau of Investigation officers have developed coping strategies to deal with exposure to illicit content. For officers who are employed in this line of work, the skill of compartmentalization is essential for sustaining their professional role [36]. Future research should examine the type and amount of illicit content exposure to increase our understanding of when and how this content becomes harmful, specifically among adults and as an occupational hazard.

MHL in the workplace continues to be an important area of focus [37]. The mediation results suggest a pathway worth noting between MHL and help-seeking for depression, although the indirect effect is modest. MHL was a partial mediator for depression, but not for anxiety or stress. The lack of mediation found between MHL and anxiety and stress could be due to the measurement timing or the cross-sectional design, which limited the ability to detect indirect effects for both anxiety and stress. Future research using longitudinal designs and alternative mediators could clarify whether these null findings reflect a true absence of mediation or are the result of methodological constraints. Unlike depression and anxiety, stress is not a medical diagnosis, but rather an automatic bodily response that is experienced after certain events. Therefore, it often does not require medical help. Stress is experienced daily and stress levels constantly fluctuate [38]. Chronic stress, on the other hand, can increase the risk for depression and anxiety [39], making it important to note that the PSS-10 instrument used in this study captured past-week perceived stress, not chronic stress.

A 2019 UK industry report by the British Interactive Media Association indicated that 28% of survey respondents experienced past-year anxiety and depression [8], a percentage that is similar to the sample in this study, 28.1% of whom reported ever having been diagnosed with a mental health condition. On the other hand, for current depression and anxiety, measured via the CESD-10 and GAD-7 scores, 29.3% of respondents were assessed as having recent symptoms of clinically significant depression and mild or greater symptoms of anxiety. These numbers are much lower than the 81% reported by the British Interactive Media Association. Differences in these numbers could be due to how the questions were formulated. This study used two validated instruments to capture symptoms, while the UK industry survey asked respondents to self-report past-year (12-month) anxiety and depression experiences.

Unfortunately, the results of this study are not directly comparable to those of the UK Biobank cohort study. It is important to note that the UK Biobank cohort study, in addition to being a longitudinal study with a UK sample, measured depression and anxiety with a single assessment tool, the Patient Health Questionnaire-4, while this US-focused study included the CESD-10 and GAD-7. Nevertheless, this study is the first to assess the relationship between IT profession–specific stressors and depression, anxiety, and stress, aiming to shed light on their associations. It also attempted to uncover the mediating role of mental health literacy between mental health conditions and help-seeking intentions.

### Limitations and Generalizability

There are a few limitations that must be noted. First, this study used a cross-sectional design, and although a mediation analysis was conducted, neither direction nor cause and effect of the relationships can be determined. The relationships found are associations and must not be regarded as causal. Furthermore, the instrument, MHL-W, used to ascertain MHL relies on self-reported level of knowledge, which might be overinflated. However, this is the only validated MHL instrument that is specific to the workplace, making it the most suitable for this type of research.

Although the sample resembled the demographic characteristics of the 357 IT professionals overall by sex (n=264, 73.9% male vs n=93, 26.1% female) [40], this sample is slightly overrepresented by White respondents (281/357, 78.7%). The 2019 US Census Bureau estimates high technology industries are comprised of primarily White (68.5%), followed by Asian (14%) IT professionals [41]. Although this sample is not representative of the demographic characteristics of the current IT sector, the goal of this study was to understand the relationship between exposures and outcome regardless of demographic information.

Despite the limitations, the results of this exploratory study are promising in that they provide insight into the types of stressors that pose a greater psychological health risk for IT professionals. Again, the possible high-risk stressors identified in this study include past-year exposure to ransomware attacks, making critical technical decisions with limited information, and dealing with leadership that is not interested in cybersecurity initiatives. These results might also be beneficial to IT leaders when considering what response plans are in place to help mitigate the psychosocial impacts these exposures may have on the IT professionals in their organizations. As has been shown previously, leadership training in mental health resources impacts employees’ willingness and actual use of those resources [42]. Furthermore, the findings highlight the important role of MHL in helping facilitate the connection between experiencing significant symptoms of depression and seeking help to address these symptoms.

### Conclusion

As the IT workforce continues to expand throughout many other sectors, thus increasing IT professional opportunities for employment, the lack of comprehensive mental health support and resources in some sectors could have negative consequences for their overall quality of life. Their health and well-being are crucial not only from a worker and an industry-level perspective, but also because of the significance of their job roles. Gaining insight into their mental health needs could facilitate the development of strategies for implementing programs aimed at improving and/or maintaining good mental health.

Before leaders can identify solutions and resources for their teams, we need leaders to be on board with supporting these teams. Given the importance of employee safety, health, and well-being, results from this study can aid IT leaders in identifying situations where IT professionals could be at an elevated risk due to workplace stressors. This awareness would enable them to proactively provide resources and support in a timely fashion. For IT-specific stressors, it is not a matter of if, but rather when, situations such as a ransomware attack will occur. Therefore, having a plan that not only focuses on the attack itself, but the human being behind the computer will be essential for promoting health among IT professionals.

## Supplementary material

10.2196/73211 Multimedia Appendix 1 Stressors experienced by the participants.

## Abbreviations

CESD-10: 10-item Center for Epidemiologic Studies Depression
CFA: confirmatory factor analysis
GAD-7: 7-item Generalized Anxiety Disorder
IT: information technology
MHL: mental health literacy
MHL-W: Mental Health Literacy Tool for the Workplace
MHSIS: Mental Help Seeking Intention Scale
PSS-10: 10-item Perceived Stress Scale
