# Supplementary material for: Associations Between IT Job Stressors and Anxiety, Depression, and Stress: Cross-Sectional Study
Source: JMIRx Med. 2026 Mar 3;7:e73211. doi: 10.2196/73211 (PMC12978965; doi:10.2196/73211)
Supplement: Multimedia Appendix 1 [file xmed-v7-e73211-s001.docx]

|  | Yes  n (%) | No  n (%) |
| --- | --- | --- |
| Ransomware attacks | 96 (26.1%) | 261 (73.1%) |
| Illicit content | 95 (26.6%) | 262 (73.4%) |
| Takedowns | 75 (21.1%) | 282 (79.0%) |
| Handling sensitive data and cybersecurity threats | 185 (51.8%) | 172 (48.2%) |
| Making critical technology decisions with limited information | 208 (58.3%) | 149 (41.7%) |
| Adapting to rapid changes in technology and business requirements | 226 (63.3%) | 131 (36.7%) |
| Pressure to solve complex technical issues | 252 (70.6%) | 105 (29.4%) |
| Constant need to stay up to date with technology | 229 (64.1%) | 128 (35.9%) |
| Dealing with unexpected system failures and outages | 245 (68.6%) | 112 (31.4%) |
| Dealing with leadership that does not wish to invest in or be inconvenienced by cybersecurity initiatives | 117 (32.8%) | 240 (67.2%) |
| Balancing security and usability | 179 (50.1%) | 178 (49.9%) |
| Working with limited resources (e.g., budget and personnel). | 206 (57.7%) | 151 (42.3%) |
|  |  | Mean (SD) |
| Total Past Year IT-Stressors | (range 1-12) | 5.41 (2.74) |
